# Supplementary material for: Venomix: a simple bioinformatic pipeline for identifying and characterizing toxin gene candidates from transcriptomic data
Source: PeerJ. 2018 Jul 31;6:e5361. doi: 10.7717/peerj.5361 (PMC6074769; doi:10.7717/peerj.5361)
Supplement: Supplemental Information 3 [file peerj-06-5361-s003.gz › FinalOutput_E-6/Snaclec_rhodocetin_subunit_delta_1/finaltree.pdf]

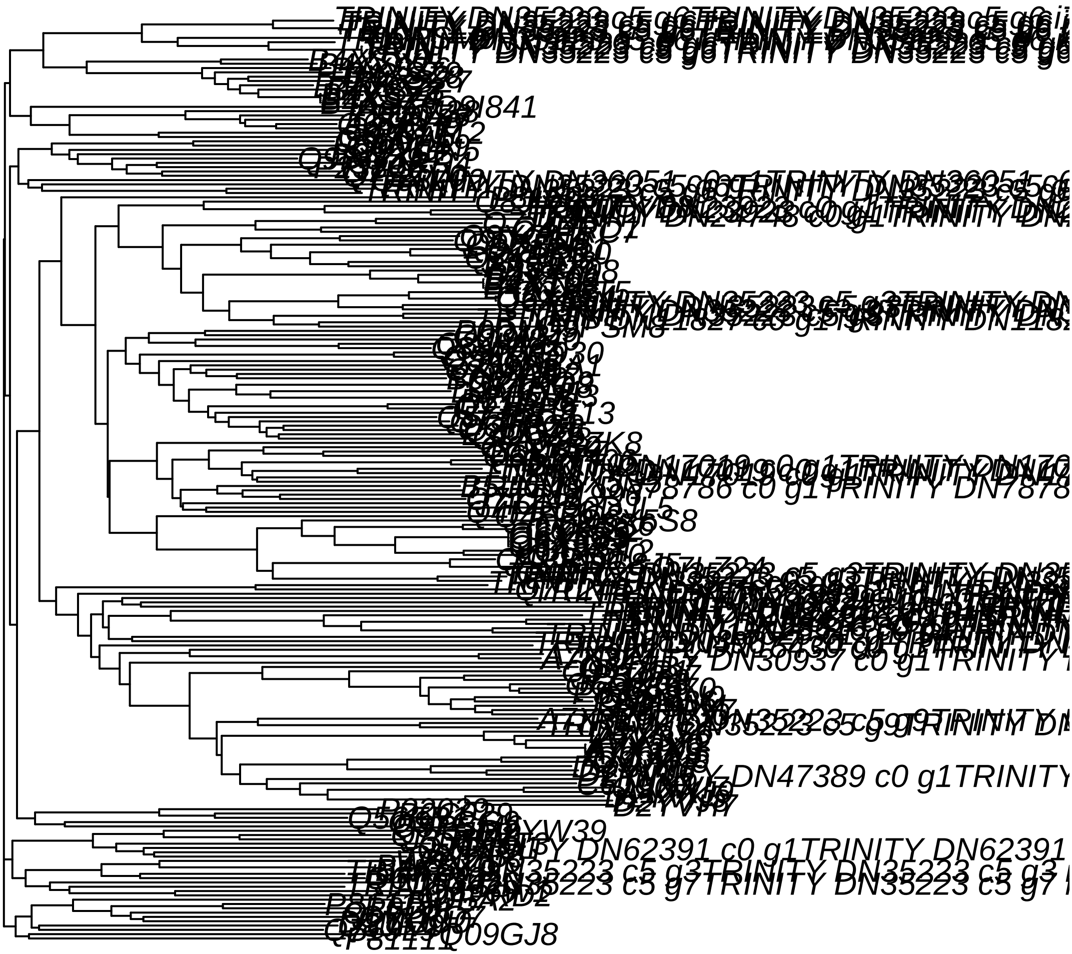
DN62391 c0 g1TRINITY DN62391
DN47389 c0 g1TRINITY
Q09GJ8
